# Review on the molecular epidemiology of sexually acquired hepatitis C virus infection in the Asia‐Pacific region

**DOI:** 10.1002/jia2.25618

**Published:** 2020-09-23

**Authors:** Chin Pok Chan, Haruka Uemura, Tsz Ho Kwan, Ngai Sze Wong, Shinichi Oka, Denise Pui Chung Chan, Shui Shan Lee

**Affiliations:** ^1^ Stanley Ho Centre for Emerging Infectious Diseases The Chinese University of Hong Kong Shatin Hong Kong; ^2^ AIDS Clinical Center National Center for Global Health and Medicine Tokyo Japan

**Keywords:** hepatitis C, molecular epidemiology, HIV, sexually transmitted diseases, genotype, Asia‐Pacific

## Abstract

**Introduction:**

Sexual acquisition has emerged as a transmission route for hepatitis C virus (HCV) of growing importance among human immunodeficiency virus (HIV)‐positive populations. In Western countries, HCV epidemics have been increasingly detected among men who have sex with men (MSM). This review describes the molecular epidemiology of sexually acquired HCV infection in the Asia‐Pacific region.

**Methods:**

A systematic search was performed on PubMed in March 2019. Either abstract or full‐text of each publication in the search results was screened for eligibility. Studies from different countries/cities involving eligible cases, who acquired HCV sexually with identified subtype, were synthesized for the evaluation of molecular epidemiology in the Asia‐Pacific region. Two large‐scale systematic reviews on the genotype distribution of HCV at a population level and among PWID were used as references for comparison.

**Results and discussion:**

Overall, 13 full‐text articles with 549 subjects originating from nine countries/cities were reviewed. A total of five genotypes and 14 subtypes were identified, dominated by subtypes 1b (23.0%), 2a (19.1%) and 3a (29.5%). A majority of the infected cases occurred in HIV‐positive MSM. In some places, notably Hong Kong, India and Indonesia, the predominant subtype in sexually acquired HCV infection in MSM was different from that circulating in the general population. Shared transmission networks between people who inject drugs (PWID) and MSM were shown in Australia and New Zealand, whereas overlapping risk elicited from a small number of subjects existed in Tokyo, Taipei and Guangxi. MSM‐specific clusters were identified in Hong Kong, Taipei and Hubei.

**Conclusions:**

The distribution of sexually acquired HCV was sparsely scattered across countries/cities in the Asia‐Pacific region. The threat of overlapping risk differed by locations, whereas transnational outbreaks remained uncommon. The paucity of information has hindered progress with comprehensive assessment in the Asia‐Pacific region, where seroprevalence of HCV among HIV‐positive MSM was relatively high.

## 1 INTRODUCTION

Hepatitis C virus (HCV), an enveloped RNA virus, is a leading cause of chronic hepatitis, cirrhosis and liver cancer [1]. In 2015, the global prevalence of chronic HCV infection was about 1%, affecting an equivalent of 71 million people, and with an estimated incidence of 1.75 million annually [2]. HCV is primarily transmitted through bloodborne contacts, notably transfusion of contaminated blood or blood products, healthcare exposure to contaminated medical equipment and shared use of syringe among people who inject drugs (PWID) [3]. In the last decade, accumulating cases of sexually acquired HCV infection suggested that high‐risk sexual behaviour has emerged to be a risk factor of increasing importance [4, 5].

In a European study, a rapid upsurge of HCV incidence among human immunodeficiency virus (HIV)‐positive men who have sex with men (MSM) was observed, from 0.09 per 100 person‐years in 1990 to 2.34 per 100 person‐years in 2007 [6]. Epidemiologically HCV is a common co‐infection in HIV‐positive persons as the two infections share a similar route of transmission. HIV‐associated immunodeficiency increases the susceptibility of the host to HCV entry and contributes to the subsequent failure of virus clearance [7]. Among MSM, engagement in unprotected anal intercourse has increased the risk of virus transmission [8]. The synergy of biological and behavioural risk factor has predisposed HIV‐positive MSM to an increased susceptibility to HCV infection.

One important cause of HCV epidemics was the widespread virus transmission in PWID. With the emergence of sexual transmission, the overlapping risk of drug injection and male‐to‐male sex has likely contributed to the rising prevalence of HCV infection among MSM, posing challenges to the development of public health intervention to achieve HCV elimination. Furthermore, extensive cross‐border transmission networks of HCV had also been uncovered in 2009 within HIV‐infected MSM in the Western countries, including the United Kingdom, the Netherlands and Germany [9]. The infection risks brought on by the complex mode of sex networking could lead to an exponential growth of the HCV epidemics in the community.

To date, seven genotypes, 67 confirmed and 20 provisionally assigned subtypes of HCV have been identified globally [10]. The continued discovery of different yet closely related variants over a short period and in diverse locations provided evidence to the phenomenon of fast‐paced virus evolution [11]. The molecular epidemiological pattern, coupled with the overlapping modes of virus transmission, suggested that the control of HCV transmission has continued to be a global challenge [11]. In the Asia‐Pacific region, information on the genetic characteristics of sexually acquired HCV is notably lacking as compared to its Western counterparts [9]. Moreover, the situation of overlapping risk and extent of HCV transmission network is yet to be systematically evaluated in this region. Against these backgrounds, this review was conducted for enhancing the understanding of molecular epidemiology of sexually acquired HCV infection in the Asia‐Pacific region to inform effective public health responses towards the elimination of the virus.

## 2 METHODS

A systematic search was performed on PubMed in March 2019. Search strategies included the inclusion of either “hepatitis C” or “HCV” in the title or abstract of a study, and in the search terms of “sexually acquired”, “men who have sex with men”, “genotype”, “phylogenetic”, “epidemiology” and their synonyms (Appendix S1). For a study to be included in this review, it must be either a cross‐sectional, cohort study or case series that has examined a representative set of samples, available in full‐text, written in English and conducted in one or more territories in the Asia‐Pacific region aligned with the definition advised by Organisation for Economic Co‐operation and Development (OECD) [12]. Because of the small number of molecular epidemiological studies on sexually acquired HCV infection, no restriction on the minimum sample size of study was imposed. Studies intentionally targeting a single genotype of HCV as research interest were excluded. PRISMA checklist was used to guide the flow of the review.

In this review, an eligible case was defined as a subject who was determined to have acquired HCV through sexual contact with an identifiable molecular subtype. Abstracts of all search results were first screened, followed by selection of studies containing eligible cases. Bibliographic reference lists of the included studies were then examined for expanding the coverage of relevant literature. For samples suspected of duplicated recruitment in multiple studies, the study with a larger number of eligible cases was retained. Characteristics of each included study were first described, including the following variables: study design, location of the study conducted, study period, source of data, study population, genomic region for sequencing, number of HCV‐viraemic and eligible samples, with HIV prevalence among the latter. The genetic profile in each study was then collated with sorting by country and city, followed by tabulation of the regional genotype distribution of HCV. Crude prevalence of each HCV genotype in the region was hence computed by summation of that in respective studies. Phylogenetic analysis performed within each included study, if available, was evaluated for the identification of shared HCV transmission network with PWID, the latter referring to those whose mode of HCV acquisition was determined by clinician/researcher to be drug injection. The presence of both cross‐border and intercity spread of HCV in the Asia‐Pacific region was also examined.

Following the systematic search, a further search on articles summarizing the prevalence of HCV genotypes in the general population and PWID in the Asia‐Pacific region was carried out. Search terms comprised “hepatitis C”, “genotype distribution”, “global”, with and without “PWID”, and their synonyms. One large‐scale study each for the general population and PWID with the most updated estimates and covering the investigated locations for sexual HCV transmission were selected. The HCV genotype distribution of these two populations in the respective countries/cities was then extracted for comparison with our main results as regards sexually transmitted HCV in the Asia‐Pacific region.

## 3 RESULTS

### 3.1 Selection of studies and study characteristics

**Figure 1:**
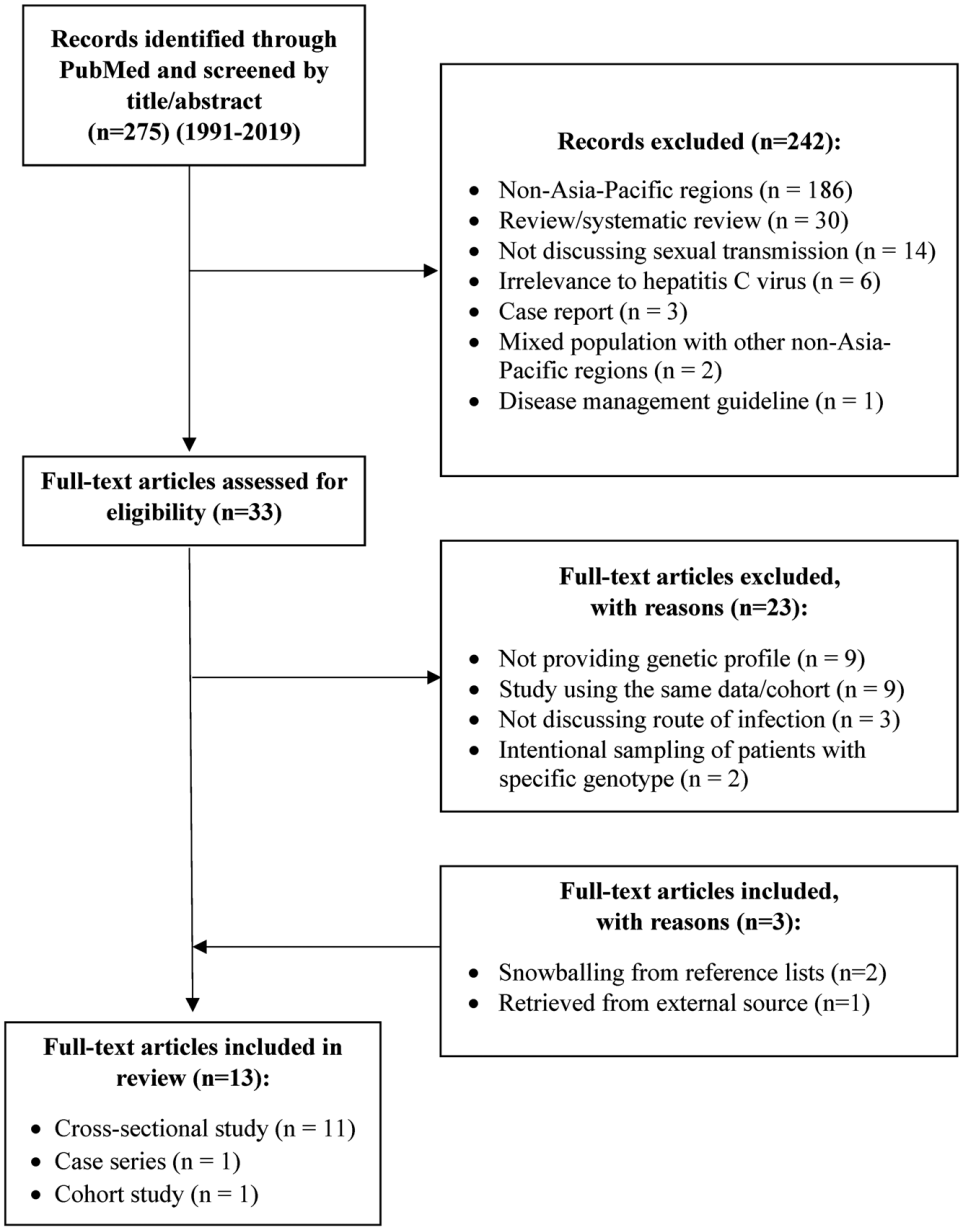
Flowchart for the selection of studies

Overall, 275 original articles examining the molecular epidemiology of sexually acquired HCV in the Asia‐Pacific region were identified, 33 full‐text of which were reviewed. Detail of the search results is shown in the flowchart (Figure 1). In total, 13 articles published from 2010 to 2019 were included, comprising 12 cross‐sectional studies and one cohort study [13, 14, 15, 16, 17, 18, 19, 20, 21, 22, 23, 24, 25]. Among them, two were multi‐centre and 11 were city‐based. The studies, all of which recruiting participants in the period of 1994 to 2016, originated from nine countries/cities, namely Australia, New Zealand, China, Hong Kong, India, Indonesia, Japan, Pakistan and Taiwan. Of 3211 HCV‐viraemic subjects in these 13 studies, 549 samples came from individuals who acquired HCV through sexual contact (including 18 through injection drug use in the presence of male‐to‐male sex), with detectable subtype identified. Of these subjects, at least 75.8% were HIV‐positive (Table 1).

**Table 1:** Characteristics of the included studies

Study (by country/city)	Study design	City	Study period	Source of data	Study population	Genomic region for sequencing	N, HCV‐viraemic^a^	N, eligible^b^, ^c^	%, HIV+
Australia & New Zealand							237	111 (18)	100
Bartlett *et al*. [13]	Cross‐sectional study	Multi‐centre	2004 to 2015	Clinic/hospital	HCV‐mono & HIV/HCV‐coinfected individuals	C/E2	237	111 (18)^d^	100
China							1209	86 (3)	≥60.5
Jiao *et al*. [14]	Cross‐sectional study	Beijing	2010 to 2011	Screening	Drug users, MSM & general population	C/E1 & NS5B	157	5	NA
Tian *et al*. [15]	Cross‐sectional study	Guangxi	2009 to 2011	Surveillance	HIV‐positive individuals	5’NC/C, C/E2, NS5B	76	17 (3)	100
Yuan *et al*. [16]	Cross‐sectional study	Guangdong	2007 to 2015	Clinic/hospital	HCV‐infected individuals	C/E1	426	17	NA
Tian *et al*. [15]	Cross‐sectional study	Henan	2009 to 2011	Surveillance	HIV‐positive individuals	5’NC/C, C/E2, NS5B	133	19	100
Zhao *et al*. [17]	Cross‐sectional study	Hubei	2007 to 2010	Surveillance	HIV‐positive individuals	C, NS5B	66	7	100
Peng *et al*. [18]	Cross‐sectional study	Hubei	2013 to 2014	Clinic/hospital	HCV‐infected individuals	C/E1, NS5B	252	10	≥90
Yang *et al*. [19]	Cross‐sectional study	Yunnan	2014	Clinic/hospital	HCV‐mono & HIV/HCV‐coinfected individuals	C/E1, NS5B	99	11	NA
Hong Kong							58	40	100
Sun *et al*. [20]	Cross‐sectional study	Hong Kong	2010 to 2016	Clinic/hospital	HIV/HCV‐coinfected individuals	NS5B	58	40	100
India							70	15	NA
Medhi *et al*. [21]	Cross‐sectional study	Multi‐centre	2005 to 2008	Clinic/hospital	Individuals with liver diseases	NS5B	70	15	NA
Indonesia							7	6	0
Hadikusumo *et al*. [22]	Cross‐sectional study	Surabaya	2012	Screening	Transgender individuals	NS5B	7	6	0
Japan							98	64	100
Ishida *et al*. [23]	Cross‐sectional study	Tokyo	1997 to 2015	Clinic/hospital	HIV‐positive individuals	Full genome	98	64	100
Pakistan							1364	78	NA
Ahmad *et al*. [24]	Cross‐sectional study	Lahore	2007 to 2009	Clinic/hospital	HCV‐infected individuals	5’NC	1364	78	NA
Taiwan							168	149	100
Sun *et al*. [25]	Cohort study	Taipei	1994 to 2010	Clinic/hospital	HIV‐positive individuals	NS5B	30	21	100
Sun *et al*. [20]	Cross‐sectional study	Taipei	2010 to 2016	Clinic/hospital	HIV/HCV‐coinfected individuals	NS5B	138	128	100
Total							3211	549 (21)	≥75.8

^a^ May not equal the sample size of the whole study

^b^ number of eligible cases (a subject who was determined to have acquired HCV through sexual contact with an identifiable molecular subtype)

^c^ number of samples with history of injection drug use in parentheses

^d^ eighteen samples from Australia who acquired HCV by injection drug use (as determined by clinician based on reported risk behaviours) with homosexual exposure for HIV acquisition were included.

As a reference on the genotype distribution of HCV in the general population, results of a study in 2015 which systematically reviewed 11 342 articles covering HCV genotype data of 115 countries were used for comparison [26]. As for PWID, another systematic review published in 2016 with genotype data from 48 studies worldwide was employed [27].

### 3.2 Genotypic characteristics of sexually acquired HCV across the Asia‐Pacific region

Overall, the sexually acquired HCV in the 549 subjects evaluated belonged to five genotypes (genotypes 1, 2, 3, 4 and 6) and 14 subtypes (subtypes 1a, 1b, 1c, 2a, 2b, 2c, 3a, 3b, 3k, 4a, 4b, 6a, 6n and 6u). Genotypes 1 (35.7%), 2 (20.4%) and 3 (32.6%) accounted for 88.7% of all cases, whereas subtypes 1b, 2a and 3a, respectively, made up 23.0%, 19.1% and 29.5% (Table 2). Other than the predominance of genotype 2 in Taipei, Hubei and Guangxi, genotype 1 or 3 was more common in all other countries/cities [15, 17, 20, 25]. Genetic diversity was noted with at least three variants recorded in each country/city, except for Surabaya, where all sequences therein belonged to only two subtypes (1b and 3k respectively) [22]. The prevalent genotypes of sexually acquired HCV were similar to those in the general population in Australia and New Zealand [13], China [14, 15, 16, 17, 18, 19], Japan [23], Pakistan [24] and Taiwan [20, 25, 26] (Table 3).

**Table 2:** Genotype distribution of sexually acquired HCV by geographic territory

Country/city	Study period	N, eligible^a^	1a, %	1b, %	2a, %	2b, %	2c, %	3a, %	3b, %	3k, %	4a, %	6a, %	6n, %	Other, %	Phylogenetic analysis	Reference
Australia & New Zealand		111	31.5					55.9						1b/2/4/6: 12.6		
Multi‐centre	2004 to 2015	111 (18)^b^	31.5					55.9						1b/2/4/6: 12.6	Yes	Bartlett *et al*. [13]
China		86	3.5	39.5	18.6			9.3	14.0			11.6	2.3	6u: 1.2		
Beijing	2010 to 2011	5		60.0					20.0			20.0				Jiao *et al*. [14]
Guangxi	2009 to 2011	17 (3)	5.9	23.5	5.9			5.9	23.5			35.3			Yes	Tian *et al*. [15]
Guangdong	2007 to 2015	17		64.7	5.9			11.8	11.8			5.9				Yuan *et al*. [16]
Henan	2009 to 2011	19	10.5	42.1	31.6			5.3					5.3	6u: 5.3	Yes	Tian *et al*. [15]
Hubei	2007 to 2010	7		28.6	28.6				28.6			14.3			Yes	Zhao *et al*. [17]
	2013 to 2014	10		40.0	60.0											Peng *et al*. [18]
Yunnan	2014	11		18.2				36.4	27.3			9.1	9.1			Yang *et al*. [19]
Hong Kong		40	7.5		2.5			87.5				2.5				
	2010 to 2016	40	7.5		2.5			87.5				2.5			Yes	Sun *et al*. [20]
India		15	40.0						6.7		33.3			1c: 20.0		
Multi‐centre	2005 to 2008	15	40.0						6.7		33.3			1c: 20.0		Medhi *et al*. [21]
Indonesia		6		33.3						66.7						
Surabaya	2012	6		33.3						66.7						Hadikusumo *et al*. [22]
Japan		64		76.6	10.9	7.8	3.1				1.6					
Tokyo	1997 to 2015	64		76.6	10.9	7.8	3.1				1.6				Yes	Ishida *et al*. [23]
Pakistan		78	17.9					67.9			12.8			4b: 1.3		
Lahore	2007 to 2009	78	17.9					67.9			12.8			4b: 1.3		Ahmad *et al*. [24]
Taiwan		149	4.0	27.5	54.4			2.7				10.7	0.7			
Taipei	1994 to 2010	21		33.3	52.4			9.5				4.8			Yes	Sun *et al*. [25]
	2010 to 2016	128	4.7	26.6	54.7			1.6				11.7	0.8		Yes	Sun *et al*. [20]
Total		549 (21)	12.2	23.0	19.1	0.9	0.4	29.5	2.4	0.7	2.9	4.9	0.5	3.5		

^a^ Number of samples with history of injection drug use in parentheses

^b^ eighteen samples from Australia who acquired HCV by injection drug use (as determined by clinician based on reported risk behaviours) with homosexual exposure for HIV acquisition were included.

**Table 3:** The two most dominant HCV subtypes in the general population, among individuals who acquired HCV through sexual contact and drug injection

Country/city	General population^a^ [26]			Individuals through sexual contact		Individuals through drug injection^b^ [27]	
	Viraemic prevalence, % (95% CI)	Viraemic population, 1000s (95% CI)	Genotype (%)	N	Genotype (%)	N	Genotype (%)
Australia	1.0 (0.7–1.0)	230 (178–244)	3 (42.2%), 1a (18.5%)	111	1a (64.2%), 3a (35.8%) [13]	134	3 (36.2%), 1a (29.0%)
New Zealand	1.0 (0.6–1.3)	48 (30–62)	1a (44.0%), 3 (35.0%)			/	Data not available
China	0.7 (0.5–0.8)	9795 (6675–10832)	1b (56.8%), 2 (15.4%)	86	1b (40.9%), 2a (17.2%) [14, 15, 16, 17, 18, 19]	126	6 (42.1%), 3 (38.1%)
Hong Kong	0.2 (0.1–0.3)	15 (6–22)	1b (62.4%), 6 (27.4%)	40	3a (79.5%), 1a (11.4%) [20]	273	6a (52.4%), 1b (38.5%) [28]
India	0.5 (0.4–0.8)	6245 (4748–10957)	3 (64.1%), 1b (16.1%)	15	1a (40.0%), 4a (33.3%) [21]	73	3 (54.7%), 1a (15.1%)/1b (15.1%)
Indonesia	0.5 (0.2–0.8)	1289 (443–2046)	1b (39.0%), 1a (25.6%)	6	3k (66.7%), 1b (33.3%) [22]	30	1a (46.6%), 3 (26.6%)
Japan	0.7 (0.3–0.8)	857 (364–1024)	1b (64.8%), 2 (34.2%)	64	1b (76.6%), 2a (10.9%) [23]	9	2 (100%)
Pakistan	3.8 (2.8–3.9)	7172 (5363–7487)	3 (79.0%), 1a (4.8%)	78	3a (67.9%), 1a (17.9%) [24]	28	2 (35.7%), 3 (28.6%)
Taiwan	2.1 (1.3–3.7)	489 (310–877)	1b (45.5%), 2 (39.5%)	149	2a (54.4%), 1b (27.5%) [20, 25]	243	1a (29.2%), 6 (28.0%)

^a^ Extracted from Global prevalence and genotype distribution of hepatitis C virus infection in 2015: a modelling study, *Lancet Gastroenterol Hepatol*, 2(3), Polaris Observatory HCV Collaborators, 161‐176, Copyright (2017), with permission from Elsevier[26]

^b^ extracted from Global genotype distribution of hepatitis C viral infection among people who inject drugs, *J Hepatol*, 65(6), Robaeys G, Bielen R, Azar DG, Razavi H, Nevens F, 1094‐1103, Copyright (2016), with permission from Elsevier [27].

### 3.3 Overlapping risk profile of HCV infection

Of the 13 studies included in this review, six (46.2%) involved phylogenetic analysis in the design [13, 15, 17, 20, 23, 25]. In these studies, the pattern of association between reported sexual risk and injection behaviours with HCV transmission differed among the places. Overlapping risk profile was shown in four out of nine countries/cities, whereas shared transmission networks between the two risk groups were phylogenetically confirmed only in the Australian‐New Zealand [13] study, as summarized in the following paragraphs.

In a multi‐centre study conducted in Australia and New Zealand during 2004 to 2015 on 237 subjects, some 41% (n = 97) acquired HCV through sexual contact while 51% (n = 121) through injection drug use [13]. Notably a total of 46% were in dyads or clusters. Three within‐group (2 sexual clusters: subtype 1a & 3a and 1 PWID cluster: subtype 1a) and 10 intergroup clusters (7 and 3 of subtype 1a and 3a respectively) were identified [13]. These clusters were largely dominated by HIV‐positive individuals, with at least one drug‐injecting MSM in each intergroup cluster [13]. Of note, some 70% of all subjects gave injection history. Those who acquired HCV through drug injection and coinfected with HIV also reported male‐to‐male sex [13]. The observed pattern suggested the presence of shared networks between MSM and PWID that had facilitated HCV transmission in Australia [13].

In a study conducted in Tokyo during 1997 to 2015, a cluster of subtype 1b comprising 13 MSM, 10 PWID and 1 heterosexual, and another of subtype 2a involving 2 MSM and 3 PWID were identified in the HIV‐positive population [23]. Noting that 16 out of these 29 clustered subjects were diagnosed with HCV after 2010, an ongoing and active transmission between MSM and PWID was implied [23].

In Taipei, two phylogenetic studies were conducted in consecutive periods in the HIV‐positive population. In 1994 to 2010, a retrospective cohort with no participants reporting a history of injection drug use revealed three pairs and four clusters inside subtypes 1b, 2a and 3a among 17 MSM and 1 heterosexual seroconverters, indicating a restricted network within HIV‐positive MSM [25]. In 2010 to 2016, another study reported the existence of two clusters of subtype 1b consisting of 14 HIV‐positive MSM and four suspected PWID [20]. An MSM‐specific cluster of subtype 6a was also recorded [20].

A study in Guangxi also put forward similar patterns of overlapping risk profile, despite unknown sex and sexual orientation of the HIV‐positive participants [15]. Inclusive of 3 reporting drug injection history, HCV sequences of 16 subjects with suspected sexually acquired HCV were mixed with 29 PWID in four clades of subtypes, which were subtypes 1a, 1b, 3b and 6a [15]. Although the bootstrap value was not available to validate the phylogenetic clustering between the two high‐risk groups, their intersection within HCV transmission network was inferred from the results [15].

By comparing the prevailing HCV variants between PWID and individuals who acquired the infection sexually, the same subtypes were only identified in Australia [13, 27]. Some similarities were also noted in India (subtype 1a) [21], Indonesia (genotype 3) [22], Japan (genotype 2) [23] and Pakistan (genotype 3) [24, 27] (Table 3).

In the remaining studies, HIV‐positive MSM‐specific HCV clusters were exclusively observed, indicating the absence of a shared network with PWID. In Hong Kong, a large monophyletic cluster of subtype 3a (n = 36) was identified in 2010 to 2016 [20]. In Hubei, a cluster of subtype 2a (n = 4) was also identified in 2007 to 2010 [17]. In the small study conducted among transgender women (n = 6) in Surabaya, subtype 3k was deduced to be a unique variant for sexually acquired HCV in Indonesia [22].

### 3.4 Intercity and cross‐border transmission of sexually acquired HCV

Only three articles had proposed possible intercity and cross‐border transmission of sexually acquired HCV, based on the analyses of a small number of samples. Five intercity clusters were described in Australia (4 and 1 of subtype 1a and 3a respectively), each involving only three to eight sequences from Brisbane, Melbourne, Perth and Sydney [13]. Intercity transmission was also suggested in China, where two sequences of subtype 2a from Hubei were shown to cluster with another two from Guangdong [17]. An incident of cross‐border transmission was speculated when a sequence from Taipei was identified within a large cluster of subtype 3a among MSM in Hong Kong [20].

## 4 DISCUSSION

This is the first review that has synthesized the current molecular epidemiology of sexually acquired HCV infection in the Asia‐Pacific region. To date, more information has come to light and confirmed the role of sexual contact as one of the major transmission routes of HCV, as well as the existence of transmission networks formed among HIV‐positive MSM [4, 7]. As revealed from the clustering patterns, local HCV networks seeded by single and multiple sources were both observed and differed by locations. A unique dominant subtype circulating among MSM existed in Hong Kong (subtype 3a) [20], India (subtype 1a) [21] and Indonesia (subtype 3k among transgender women) [22, 26]. Probably due to founder effect, the founding MSM in these places might have isolated from the large infected population and established a new gene pool, echoing the HCV‐4d outbreaks among MSM in Europe [29, 30, 31], where genotype 1 was predominant [9, 32]. Elsewhere in the Asia‐Pacific, the genotypes harboured among those who acquired HCV through sexual contacts appeared to be a replicated subset of that circulating in the general population.

In the assessment of the overlapping risk of MSM and PWID in the Asia‐Pacific region, two different patterns were observed. In Australia and New Zealand, the extensive co‐clustering of HCV between MSM and PWID concurred with the results of another study conducted in 2009, which identified two intergroup clusters within the domestic transmission network in Australia [9, 13]. Considering the substantial overlap between the two risk groups, a majority of the clustered individuals in both studies had acquired HCV probably through “slamming”, the term referring to sexualized use of recreational drugs (chemsex) by injection among MSM in recent years [33, 34]. Similar to the setting of HIV epidemics, drug‐injecting MSM appeared to constitute the overlapping risk profile of HCV [35]. This was further supported by the similar set of prevalent genotypes among MSM and PWID in Australia and New Zealand [13, 27]. On the other hand, limited co‐clustering of MSM and PWID was identified in Taipei [25] and Tokyo [23], and the coexistence of the same clade was noted in Guangxi [15]. Given the disclosure of both risk behaviours by a small proportion of clustered individuals in these reported studies, HCV might have spilled‐over from PWID to MSM or vice versa. These putative and limited intergroup networks could have been derived in the presence of a small number of drug‐injecting MSM instead of non‐MSM PWID. However, the limited availability of published research did not allow us to conclude definitively the occurrence of any intergroup spread in countries/cities outside Australia and New Zealand. Similar to Europe where there were dissimilar subsets of leading HCV subtypes between MSM and PWID (subtype 1a & 4d for MSM versus genotype 3 for PWID) [9, 27], distinctive sexual transmission networks were also seen in locations in the Asia‐Pacific like China [14, 15, 16, 17, 18, 19] and Hong Kong [20, 28]. In perspective, evidence suggests the existence of the widespread overlapping risk of HCV in Australia and New Zealand but not in Asian countries. Interpretation of HCV molecular epidemiology is, however, made more difficult by the co‐existence of two different transmission routes, which are male‐to‐male sex and drug injection. Since the two populations, MSM and PWID, are not mutually exclusive, it might be hard to dichotomize study participants for comparison if they reported both risk behaviours. Nevertheless, identification on transmission pattern between properly defined PWID and MSM should be strengthened, considering the presence of PWID‐dominant subtype 6a cluster among MSM in Taipei [20], and the mounting dual risk among transgender populations in South Asia [36].

In comparison to Europe where transnational epidemics of sexually acquired HCV were detected, the molecular epidemiology in the Asia‐Pacific region does not show signs of the parallel existence of an international outbreak [9, 37]. By contrasting the genotype profiles of different countries/cities in our review, the high degree of heterogeneity of the leading subtypes, including subtype 1a, 1b, 2a, 3a and 3k, indicated the generally discrete pattern of local transmission of HCV with little spread between territories. The diversity, that sharply contrasted the shared genotypes across European territories, could be the results of the geographical separation between countries in the Asia‐Pacific region, as well as the relatively low connectedness among Asian communities [9, 37, 38]. The molecular evidence reported in the included studies were too slim to confirm the existence of any cross‐border and intercity transmission networks of HCV [13, 17, 20]. The identification of a related sequence between Taiwan and Hong Kong could have been driven by occasional travels without the growth of a sustained transmission network among MSM [20]. For the related cases reported in China, the delineation of the cluster could be due to the lack of discrimination, given the utilization of the conserved core region for genotyping [17, 39]. The inclusion of Australian samples from different cities in the same cluster was not robust enough to assert a genuine transmission event, since the founding subtypes in different regions of Australia were largely identical [13, 40]. Demonstration of HCV spread between regions could only be confirmed if a larger intercity cluster is detected. More data at city and national level are required to understand the scale and transmission dynamics of within‐territory and cross‐border HCV networks. Therefore, currently there is no evidence to support the overlap of HCV transmission networks among MSM between territories in the Asia‐Pacific region and with other continents. Yet, in light of the escalating popularity of gay party along with the growing ease of transportation worldwide, the potential risk of cross‐border HCV transmission should not be underestimated [41].

Overall, the growth of HCV epidemics among MSM in the Asia‐Pacific region, alongside the lack of information, is a cause for concern. With reference to a systematic review and meta‐analysis, the HCV seroprevalence among HIV‐positive MSM, based on ten included studies in Australia, China, Japan and Taiwan, averaged 9.42% (range: 1.2% to 24.3%), which was higher than that in Europe (n = 21, 7.7%, 2.6% to 18%) but slightly lower than North America (n = 9, 10.7%, 4.3% to 17.9%) [8]. In comparison to the global HCV seroprevalence among the general population varying between 0.5% and 2.3%, considerably higher estimates were also recorded in some Asian countries: Myanmar (1.0%), Laos (1.1%), Indonesia (2.1%), Cambodia (2.3%), Thailand (2.7%) and Vietnam (6.1%) [3, 42]. Furthermore, the high prevalence of HIV (2% to 29%) among the Southeast Asian sex workers, transgender people and MSM has also rendered them to an enhanced risk of sexually acquired HCV infection [7, 43]. At present, there is a paucity of published molecular epidemiological data, at both city and country level, on HCV infection resulting from sexual transmission in the Asia‐Pacific region, despite the existence of epidemic risks. From a regional perspective, it is important to conduct molecular studies to illustrate the evolution of HCV, infer phylogenetic relationships and identify sexual networks. The functioning of collaborative HCV cohorts with a regional context might be one of the solutions in strengthening preparedness against HCV epidemics, through the establishment of a regional database, and facilitating epidemiologic analyses [44].

There are several limitations in this study. First, only published data from nine territories in the Asia‐Pacific region could be reviewed which might undermine the generalizability of the synthesized results. The limited number of HCV samples in each territory, that might be disproportionate to the true nationwide or city‐wide population prevalence, could lead to skewed results on the overall genotype distribution, which should be interpreted with caution. Second, the differentiation of transmission patterns between MSM versus non‐MSM and by HIV status was technically difficult. Since the baseline characteristics corresponding to each genotyped sequence were not specifically profiled in every study, the epidemiologic patterns could only be jointly evaluated. The same situation also applied to the differentiation between MSM‐PWID and conventionally defined PWID, which might also make the interpretation on overlapping risks difficult. Third, the incompleteness of sequence data and the unavailability of the accession numbers of the sexually acquired HCV sequences in most studies did not allow us to validate the clustering results. The differences in the genomic region used for sequencing in multiple studies had further restricted our evaluation and interpretation on the phylogenies and their implications.

## 5 CONCLUSIONS

In the Asia‐Pacific region, the genotype distribution of sexually acquired HCV is scattered across cities and countries. Currently, shared transmission network between MSM and PWID was noted only in Australia and New Zealand, while overlapping risk elicited from a small number of subjects existed in other parts of the region. Nevertheless, these networks were more likely to have been formed by drug‐injecting MSM instead of heterosexual PWID. Molecular evidence was minimal to reveal any cross‐border and intercity transmission network, and that the occurrence of regional outbreaks could not be demonstrated. The scarcity of molecular epidemiological information has hindered the progress with conducting comprehensive assessment in the Asia‐Pacific region, where seroprevalence of HCV among HIV‐positive MSM was relatively high.

## COMPETING INTERESTS

The authors have no competing interests to declare for this study.

## AUTHOR’S CONTRIBUTIONS

CP Chan wrote the first draft of manuscript with input from all authors. H Uemura, S Oka and DPC Chan contributed to the interpretation and verification of results. TH Kwan and NS Wong helped revise the manuscript. SS Lee conceived the original idea and supervised the study. All authors provided critical feedback and contributed to the final manuscript.

## Supporting information


**Appendix S1.** Search strategy for studies on sexually acquired HCV infection

## References

[1] Rosen HR . Chronic hepatitis C infection. N Engl J Med. 2011;364(25):2429–38. 21696309 10.1056/NEJMcp1006613

[2] World Health Organization . Global Hepatitis Report 2017 [cited 2019 Aug 8]. Available from: https://apps.who.int/iris/bitstream/handle/10665/255016/9789241565455‐eng.pdf

[3] World Health Organization . Hepatitis C [cited 2019 Aug 8]. Retrieved from: https://www.who.int/news‐room/fact‐sheets/detail/hepatitis‐c

[4] Chan DP , Sun HY , Wong HT , Lee SS . Sexually acquired hepatitis C virus infection: a review. Int J Infect Dis. 2016;49:47–58. 27270138 10.1016/j.ijid.2016.05.030

[5] Wong NS , Lee CK , Ng SC , Wong HK , Chan DPC , Lee SS . Prevalence of hepatitis C infection and its associated factors in healthy adults without identifiable route of transmission. J Viral Hepat. 2018;25:161–70. 29032634 10.1111/jvh.12804

[6] Van der Helm JJ , Prins M , del Amo J , Bucher HC , Chene G , Dorrucci M , et al. The hepatitis C epidemic among HIV‐positive MSM: incidence estimates from 1990 to 2007. AIDS. 2011;25:1083–91. 21537114 10.1097/QAD.0b013e3283471cce

[7] Nijmeijer BM , Koopsen J , Schinkel J , Prins M , Geijtenbeek TB . Sexually transmitted hepatitis C virus infections: current trends, and recent advances in understanding the spread in men who have sex with men. J Int AIDS Soc. 2019;22 Suppl 6:e25348. 31468692 10.1002/jia2.25348 PMC6715947

[8] Jordan AE , Perlman DC , Neurer J , Smith DJ , Des Jarlais DC , Hagan H . Prevalence of hepatitis C virus infection among HIV+ men who have sex with men: a systematic review and meta‐analysis. Int J STD AIDS. 2017;28(2):145–59. 26826159 10.1177/0956462416630910 PMC4965334

[9] van de Laar T , Pybus O , Bruisten S , Brown D , Nelson M , Bhagani S , et al. Evidence of a large, international network of HCV transmission in HIV‐positive men who have sex with men. Gastroenterology. 2009;136(5):1609–17. 19422083 10.1053/j.gastro.2009.02.006 PMC4260925

[10] Smith DB , Bukh J , Kuiken C , Muerhoff AS , Rice CM , Stapleton JT , et al. Expanded classification of hepatitis C virus into 7 genotypes and 67 subtypes: updated criteria and genotype assignment web resource. Hepatology. 2014;59(1):318–27. 24115039 10.1002/hep.26744 PMC4063340

[11] Echeverría N , Moratorio G , Cristina J , Moreno P . Hepatitis C virus genetic variability and evolution. World J Hepatol. 2015;7(6):831–45. 25937861 10.4254/wjh.v7.i6.831 PMC4411526

[12] Organisation for Economic Cooperation and Development . Health at a Glance: Asia/Pacific 2018 [cited 2019 Sep 18]. Available from: https://www.oecd‐ilibrary.org/docserver/health_glance_ap‐2018‐en.pdf?expires=1569402852&id=id&accname=ocid177302&checksum=82ACE356694C49FC70C5CB56505B3D52

[13] Bartlett SR , Applegate TL , Jacka BP , Martinello M , Lamoury FM , Danta M , et al. A latent class approach to identify multi‐risk profiles associated with phylogenetic clustering of recent hepatitis C virus infection in Australia and New Zealand from 2004 to 2015. J Int AIDS Soc. 2019;22(2):e25222. 30746864 10.1002/jia2.25222 PMC6371014

[14] Jiao Y , Zhang X , Wang C , Li L , Liu J , Bar KJ , et al. Hepatitis C virus subtype and evolution characteristic among drug users, men who have sex with men, and the general population in Beijing, China. Medicine. 2016;95:e2688. 26871798 10.1097/MD.0000000000002688 PMC4753893

[15] Tian D , Li L , Liu Y , Li H , Xu X , Li J . Different HCV genotype distributions of HIV‐infected individuals in Henan and Guangxi, China. PLoS One. 2012;7:e50343. 23226265 10.1371/journal.pone.0050343 PMC3511438

[16] Yuan G , Liu J , Hu C , Huang H , Qi M , Wu T , et al. Genotype distribution and molecular epidemiology of hepatitis C virus in guangzhou, china: predominance of genotype 1b and increasing incidence of genotype 6a. Cell Physiol Biochem. 2017;43(2):775–87. 28950254 10.1159/000481561

[17] Zhao R , Peng J , Tang L , Huang H , Liu M , Kong W , et al. Epidemiological distribution and genotype characterization of hepatitis C virus and HIV co‐infection in Wuhan, China, where the prevalence of HIV is low. J Med Virol. 2013;85(10):1712–23. 23868809 10.1002/jmv.23650

[18] Peng J , Lu Y , Liu W , Zhu Y , Yan X , Xu J , et al. Genotype distribution and molecular epidemiology of hepatitis C virus in hubei, central China. PLoS One. 2015;10:e0137059. 26325070 10.1371/journal.pone.0137059 PMC4556612

[19] Yang L , Jiang C , Hu S , Diao Q , Li J , Si W , et al. Evolving diversity of hepatitis C viruses in Yunnan Honghe, China. Int J Mol Sci. 2016;17(3):403. 26999127 10.3390/ijms17030403 PMC4813258

[20] Sun HY , Uemura H , Wong NS , Chan DP , Wong BC , Lin PH , et al. Molecular epidemiology of acute HCV infection in HIV‐positive patients from Hong Kong, Taipei, Tokyo. Liver Int. 2019;39(6):1044–51. 30770636 10.1111/liv.14073

[21] Medhi S , Goswami B , Das AK , Singh TB , Husain SA , Sehgal A , et al. New insights into hepatitis C virus infection in the tribal‐dominant part of Northeast India. Arch Virol. 2012;157(11):2083–93. 22791109 10.1007/s00705-012-1374-z

[22] Hadikusumo AA , Utsumi T , Amin M , Khairunisa SQ , Istimagfirah A , Wahyuni RM , et al. High Rates of Hepatitis B Virus (HBV), Hepatitis C Virus (HCV), and Human Immunodeficiency Virus Infections and Uncommon HBV Genotype/Subtype and HCV Subtype Distributions among Transgender Individuals in Surabaya, Indonesia. Jpn J Infect Dis. 2016;69(6):493–9. 27000450 10.7883/yoken.JJID.2015.384

[23] Ishida Y , Hayashida T , Sugiyama M , Tsuchiya K , Kikuchi Y , Mizokami M , et al. Full‐genome analysis of hepatitis C virus in Japanese and Non‐Japanese patients coinfected with HIV‐1 in Tokyo. J Acquir Immune Defic Syndr. 2019;80(3):350–7. 30550489 10.1097/QAI.0000000000001919

[24] Ahmad W , Ijaz B , Javed FT , Jahan S , Shahid I , Khan FM , et al. HCV genotype distribution and possible transmission risks in Lahore. Pakistan. World J Gastroenterol. 2010;16(34):4321–8. 20818816 10.3748/wjg.v16.i34.4321 PMC2937113

[25] Sun HY , Chang SY , Yang ZY , Lu CL , Wu H , Yeh CC , et al. Recent hepatitis C virus infections in HIV‐infected patients in Taiwan: incidence and risk factors. J Clin Microbiol. 2012;50(3):781–7. 22189113 10.1128/JCM.06014-11 PMC3295121

[26] Polaris Observatory HCV Collaborators . Global prevalence and genotype distribution of hepatitis C virus infection in 2015: a modelling study. Lancet Gastroenterol Hepatol. 2017;2(3):161–76. 28404132 10.1016/S2468-1253(16)30181-9

[27] Robaeys G , Bielen R , Azar DG , Razavi H , Nevens F . Global genotype distribution of hepatitis C viral infection among people who inject drugs. J Hepatol. 2016;65(6):1094–103. 27520879 10.1016/j.jhep.2016.07.042

[28] Chan D , Lee SS , Lee KC . The effects of widespread methadone treatment on the molecular epidemiology of hepatitis C virus infection among injecting drug users in Hong Kong. J Med Virol. 2011;83:1187–94. 21567422 10.1002/jmv.22099

[29] Vogel M , van de Laar T , Kupfer B , Stellbrink HJ , Kümmerle T , Mauss S , et al. Phylogenetic analysis of acute hepatitis C virus genotype 4 infections among human immunodeficiency virus‐positive men who have sex with men in Germany. Liver Int. 2010;30(8):1169–72. 20633101 10.1111/j.1478-3231.2010.02305.x

[30] de Bruijne J , Schinkel J , Prins M , Koekkoek SM , Aronson SJ , van Ballegooijen MW , et al. Emergence of hepatitis C virus genotype 4: phylogenetic analysis reveals three distinct epidemiological profiles. J Clin Microbiol. 2009;47(12):3832–38. 19794040 10.1128/JCM.01146-09 PMC2786681

[31] Visseaux B , Hué S , Le Hingrat Q , Salmona M , Lebourgeois S , Delaugerre C , et al. Phylogenetic investigation of HCV‐4d epidemic in Paris MSM HIV population reveals a still active outbreak and a strong link to the Netherlands. Clin Microbiol Infect. 2020 26 6:785.e1–785.e4. 32035235 10.1016/j.cmi.2020.01.034

[32] Provine WB . Ernst Mayr: genetics and speciation. Genetics. 2004;167(3):1041–6. 15280221 10.1093/genetics/167.3.1041 PMC1470966

[33] Fridae . Self‐reported HIV prevalence, HIV testing history and risk behaviour among MSM in 17 countries in Southeast and East Asia: Comparing results from two large scale, multi‐language online surveys in 2009 and 2010 [cited 2019 Sep 12]. Available from: http://www.fridae.asia/download/aimss_stats_A4.pdf

[34] Bui H , Zablotska‐Manos I , Hammoud M , Jin F , Lea T , Bourne A , et al. Prevalence and correlates of recent injecting drug use among gay and bisexual men in Australia: results from the FLUX study. Int J Drug Policy. 2018;55:222–30. 29429864 10.1016/j.drugpo.2018.01.018

[35] Martin NK , Vickerman P , Hickman M , Patterson TL , Rand E , Abramovitz D , et al. Overlapping substance using high‐risk groups and infectious diseases: how dynamic modelling can evaluate risk and target HIV prevention. Addiction. 2016;111(9):1512–15. 27075692 10.1111/add.13338 PMC4983200

[36] Deacon RM , Mooney‐Somers J , Treloar C , Maher L . At the intersection of marginalised identities: lesbian, gay, bisexual and transgender people's experiences of injecting drug use and hepatitis C seroconversion. Health Soc Care Community. 2013;21(4):402–10. 23465052 10.1111/hsc.12026

[37] Salazar‐Vizcaya L , Kouyos RD , Metzner KJ , Caraballo Cortes K , Böni J , Shah C , et al. Changing trends in international versus domestic HCV transmission in HIV‐positive men who have sex with men: a perspective for the direct‐acting antiviral scale‐up era. J Infect Dis. 2019;220(1):91–9. 30759225 10.1093/infdis/jiz069 PMC6548898

[38] Schnell G , Krishnan P , Tripathi R , Beyer J , Reisch T , Irvin M , et al. Hepatitis C virus genetic diversity by geographic region within genotype 1–6 subtypes among patients treated with glecaprevir and pibrentasvir. PLoS One. 2018;13:e0205186. 30286205 10.1371/journal.pone.0205186 PMC6171933

[39] Lamoury FM , Jacka B , Bartlett S , Bull RA , Wong A , Amin J , et al. The influence of hepatitis C virus genetic region on phylogenetic clustering analysis. PLoS One. 2015;10:e0131437. 26192190 10.1371/journal.pone.0131437 PMC4507989

[40] Matthews GV , Pham ST , Hellard M , Grebely J , Zhang L , Oon A , et al. Patterns and characteristics of hepatitis C transmission clusters among HIV‐positive and HIV‐negative individuals in the Australian trial in acute hepatitis C. Clin Infect Dis. 2011;52(6):803–11. 21282185 10.1093/cid/ciq200 PMC3106259

[41] Cheung DH , Lim SH , Guadamuz TE , Koe S , Wei C . The potential role of circuit parties in the spread of HIV among men who have sex with men in asia: a call for targeted prevention. Arch Sex Behav. 2015;44(2):389–97. 25104105 10.1007/s10508-014-0339-6 PMC4320983

[42] Doan TQ . Hepatitis C in Developing Countries in Southeast Asia In: Kamal SM, ed. Hepatitis C in Developing Countries ‐ Current and Future Challenges. 1st ed. Cambridge: Academic Press; 2018 p. 87–104.

[43] Pendse R , Gupta S , Yu D , Sarkar S . HIV/AIDS in the South‐East Asia region: progress and challenges. J Virus Erad. 2016;2 Suppl 4:1–6. 10.1016/S2055-6640(20)31092-X PMC5353351 28303199

[44] Grebely J , Morris MD , Rice TM , Bruneau J , Cox AL , Kim AY , et al. Cohort profile: the international collaboration of incident HIV and hepatitis C in injecting cohorts (InC3) study. Int J Epidemiol. 2013;42(6):1649–59. 23203695 10.1093/ije/dys167 PMC3887561

