# Supplementary material for: Review on the molecular epidemiology of sexually acquired hepatitis C virus infection in the Asia‐Pacific region
Source: J Int AIDS Soc. 2020 Sep 23;23(9):e25618. doi: 10.1002/jia2.25618 (PMC7511596; doi:10.1002/jia2.25618)
Supplement: Supplementary file 1 — Appendix S1. Search strategy for studies on sexually acquired HCV infection [file JIA2-23-e25618-s001.docx]

**Appendix S1. Search strategy for studies on sexually acquired HCV infection**

Database searched: PubMed

Date last searched: 19 Mar 2019

Number of results: 275 (published between 1991 and 2019)

1. hepatitis c [title/abstract]
2. HCV [title/abstract]
3. 1 or 2
4. intercourse
5. sexual$
6. permucosal$
7. homosexual$
8. gay
9. MSM
10. men who have sex with men
11. 4 or 5 or 6 or 7 or 8 or 9 or 10
12. genotype
13. phylogeny$
14. cluster$
15. molecular
16. 12 or 13 or 14 or 15
17. epidemic
18. epidemiology
19. incidence
20. prevalence
21. 17 or 18 or 19 or 20
22. regression
23. English [lang]
24. 3 and 11 and 16 and 21
25. 24 not 22
26. 25 and 23
